# Negative impact of gestational diabetes mellitus on progress of pelvic floor muscle electromyography activity: Cohort study

**DOI:** 10.1371/journal.pone.0223261

**Published:** 2019-11-07

**Authors:** Caroline B. Prudencio, Marilza V. C. Rudge, Fabiane A. Pinheiro, Carlos I. Sartorão Filho, Sthefanie K. Nunes, Cristiane R. Pedroni, Baerbel Junginger, Angélica M. P. Barbosa

**Affiliations:** 1 Department of Gynecology and Obstetrics, Botucatu Medical School, São Paulo State University (Unesp), Universidade Estadual Paulista (UNESP), Botucatu, São Paulo, Brazil; 2 Department of Physiotherapy and Occupational Therapy, School of Philosophy and Sciences, São Paulo State University (Unesp), Universidade Estadual Paulista (UNESP), Marilia, São Paulo, Brazil; 3 Gynecology Department, Charité University Hospital, Berlin, Germany; Medical College of Wisconsin, UNITED STATES

## Abstract

**Background and objective:**

Pelvic floor muscles are involved in postural stability, in maintenance intra-abdominal pressure, and on mechanical support for pelvic organ. Gestational Diabetes Mellitus’ (GDM) pregnancies complicated by fetal macrosomia, large placenta and polyhydramnios contribute for abrupt and intense increase in maternal intra-abdominal pressure. Our objective was analyze the impact of GDM on pelvic floor muscle (PFM) electromyography (EMG) activity progress from 24–30 to 36–38 weeks of gestation. We conducted a prospective cohort study. PFM EMG was performed in nulliparous or primiparous women with one previous elective cesarean delivery and with or not GDM diagnosed by the American Diabetes Association criteria. A careful explanation of the muscle anatomy and functionality of the PFM was given before EMG assessment. The outcome measures were PFM recruitment and progress from 24–30 to 36–38 weeks of gestation analyzed by the normalized root mean square (RMS) during rest-activity, fast and hold pelvic floor muscle contraction.

**Results:**

Fifty-two pregnant women were assigned to 2 groups: the GDM (n = 26) and normoglycemic (NG) (n = 26). The demographic and obstetric data showed homogeneity between the groups. PFM activity progress was decreased in rest-activity (*P* = 0.042) and hold contraction (P = 0.044) at 36–38 weeks of gestation in the GDM group relative to that in the NG group.

**Conclusion:**

GDM group showed a progressive decrease in EMG-PFM activity during rest-activity and hold contractions from 24–30 to 36–38 weeks of gestation.

## Introduction

Maternal risk and perinatal outcome are widely researched during pregnancy complicated by hyperglycemic disorders. [1,2] Nevertheless, another relevant and less investigated aspect involved in hyperglycemic pregnancies is the urinary disorders that remaining unanswered. Few studies have been published about the gestational diabetes mellitus (GDM) influence on pelvic floor muscles (PFM) function, although there are evidences that GDM during pregnancy was responsible to increase urinary incontinence (UI) rates and to decrease PFM squeeze pressure even 2 years after C-section. [3,4] Clinical evidence crossing UI, GDM and pelvic floor muscle dysfunction (PFMD) supported experimental studies to investigate possible pathological changes on muscular tissues and rats was choose because the striated urethral muscle distribution and neuroanatomy are similar to human.

Changes in urethral striated muscles in severe diabetes and mild diabetic pregnant rats have demonstrated atrophy, thinning, disorganization, rupture of muscle fibers, and loss of specific fiber types from normal anatomical locations, all of which are characteristics of diabetic myopathy.[5,6] Further changes in the distribution of the extracellular matrix, such as increased interstitial collagen, lipids, and mitochondria, have been observed in striated muscle.[5,6] Furthermore, metabolic defects of substrates involved in adenosine triphosphate (ATP) formation, protein turnover, lipolysis, and lipogenesis, such as neural lesions,[7–9] was described in GDM. [10,11]

Therefore, to confirm experimental findings in clinical studies, methodological and ethical concerns might be faced because PFM biopsy is required during delivery. In humans, EMG is an indirect tool to verify neuromuscular integrity therefore it is a methodological solution to access possible neural and muscular disorders caused by GDM. [12] It is a tool adopted in studies with no-pregnancy hyperglycemic disturbance to evaluate PFM function and allowed to detect motor control disturb and PFM function decrement.[13]

This present clinical research was based on findings from previous experimental results and now in a "bench to bedside" step of translational approach, intends to clarify the relationship of PFM function and GDM pregnancy. This is the first study to evaluate the influence of GDM during pregnancy on PFM recruitment and its progression from second to third trimester. [14,15] Therefore, the research question for this prospective cohort study was:

Does gestational diabetes mellitus (GMD) alter the pelvic floor muscle recruitment progression from 24–30 to 36–38 weeks of gestation in pregnant women?

## Method

### Design

This prospective cohort study was approved by the Institutional Ethical Committee of Botucatu Medical School of São Paulo State University (Protocol Number 972.104). The protocol was explained to voluntary participants. They were informed that they could withdraw their consent at any time during cohort. After learning all procedures, all subjects provided a written consent to the study. The Helsinki Declaration on human experimentation guidelines was respected.

### Participants, therapists, centres

All participants followed the conventional prenatal protocol of the Brazilian health system at the Perinatal Diabetes Research Center (PDRC) of Botucatu Medical School/UNESP/Brazil, between 2015 and 2016. A single trained physiotherapist with 4 years of experience in PFM evaluation performed the physical examination. The threshold to compose the study groups was the 75g oral glucose tolerance test (OGTT). All pregnant women in screening and diagnostic phase at 24–30 weeks of gestation underwent OGTT. According to ADA criteria(2015), [3] pregnant who underwent fasting ≥92 mg/dL or 1 hour ≥180 mg/dL or 2 hours ≥153 mg/dL were allocated to the GDM group. The participants who had lower values assigned the normoglycemic group (NG). The inclusion criteria were: pregnant women between 24–30 weeks of gestation; singleton pregnancy; 18–40 years of age; they had to be able to contract PFM, have not previously or during pregnancy performed PFM training or any additional musculoskeletal PFM treatment. The exclusion criteria were clinical diabetes (type I or II or overt diabetes in previous pregnancy), urinary incontinence, >2 pregnancies, previous prolapse or incontinence surgery, no understanding of the command to contract PFM, neurological diseases, diagnosis of genital prolapse, cervical isthmus incompetence, smoking, participants who withdraw their consent during cohort, preterm birth and abortion.

Concerning glycemic control, following a diagnosis of GDM, all pregnant women from GDM group have received information from the health team noting that the normalization of maternal glucose was essential to maternal and fetal health as well as of short and long-term effects on the mother’s health and of her offspring. Blood glucose control was performed by Glucose Meter and GDM group included women who presented strict glycemic control after GDM diagnosis. [2]

### Sample size estimation

Sample size was obtained by G*Power software using values of hold contraction at 36–38 weeks of gestation from our previous pilot study. Determining a sample effect of 0.846, two-sided α of 0.05, and a power of 80%, 23 pregnant women in each group were required in order to detect differences.

### Intervention

Pregnant women who agreed to participate were contacted and invited at 24–30 weeks of gestation and rescheduled at 36–38 weeks of gestation to repeat the same initial procedures. Data collected from hospital records were confirmed by the patient, and body mass index (BMI) was measured at both time points (calculated as weight [kg]/height^2^ [m]).

Bladder emptying was requested. Participants were examined in the supine position with their lower limbs flexed with feet on the stretcher, and information about the anatomical position and possible movement of the PFM was obtained to avoid the use of adductor and/or gluteus, hip movements or expulsive movements. To be considered correct the PFM contraction, a vaginal palpation was performed, and a PFM contraction was requested by giving the verbal instruction “squeeze the vaginal muscle and hold as if you were holding urine.” The contraction was considered to be correct if the examiner felt an inward pressure and/or upward traction in palpation. The participants had 3 chances to perform maximal voluntary contraction (1 second to contract and relax afterwards) and 3 chances to perform hold contractions (1 to 10 seconds holding and relaxing), respectively, simulating the steps of the EMG test performed later on. Contraction of the adductor, gluteus, hip movements, and expulsion movements was discouraged and rectified.[16,17] Five minutes of resting was performed before EMG.

The EMG measurement was performed by using a two-channel device (Miotool 200 Uro; Porto Alegre, Brazil) with a gain of 1000, 14-bit A/D converter, input impedance of 10^10^ Ohm/2 pF, CMRR at 126 dB common, band-pass filter of 20–500 Hz, and 2 kHz sampling rate. EMG activity of PFM was recorded by using a vaginal probe sensor with two opposite stainless steel electrodes (85 x 25 mm) positioned on both vaginal sidewalls coupled to an active differential sensor with ring connection and 100 times gain. A water-soluble gel was used to introduce the probe into the vaginal canal. Skin was prepared by using a 70% ethanol solution to fix the reference electrode on the ulna’s styloid process.[18,19]

**Fig 1:**
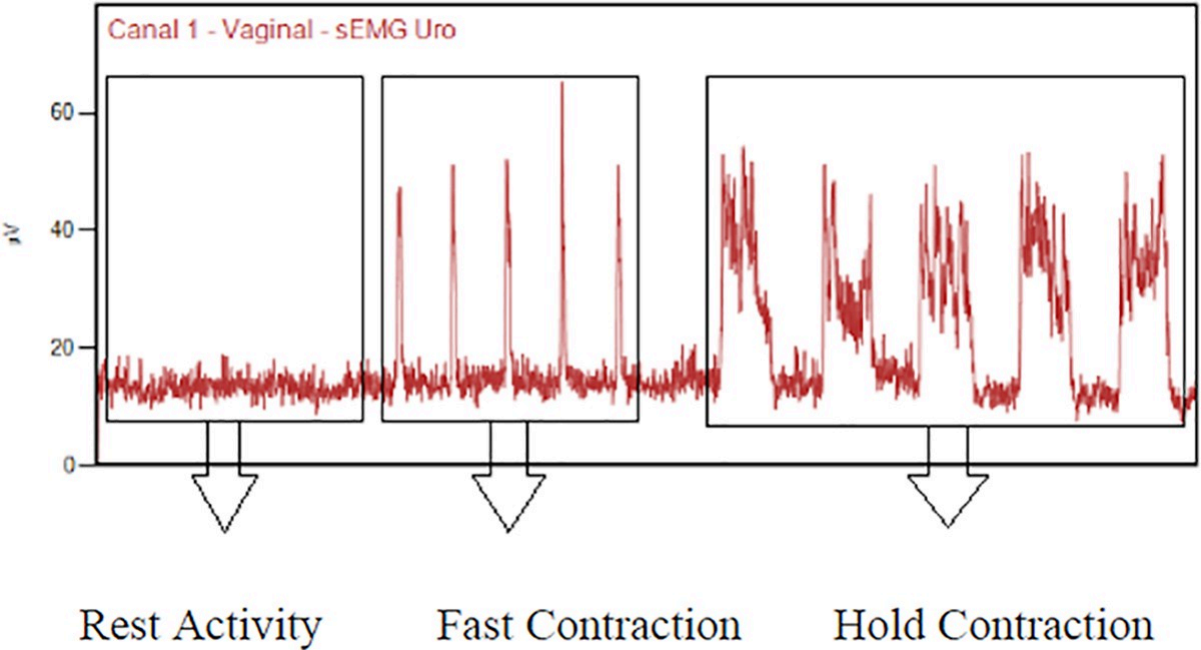
Modified Glazer Protocol plots showing rest activity and fast and hold contraction.

For the EMG recordings, modified Glazer protocol was used to verify muscle activity during rest-activity, fast and hold contractions.[20,21] The sequence consisted of 3 segments: 1) to assess lower basal activity of PFM a 60 second preliminary resting baseline was defined as the rest-activity; 2) five fast contractions or “flicks,” each preceded by a 10 second rest period, were defined as fast contractions; and 3) five repetitions of 10 second contractions, each contraction preceded by a 10 second rest period, were defined as hold contractions (Fig 1). [20,21] Women were instructed about the sequence and need to contract the PFM immediately when verbally instructed by the researcher. The same evaluation sequence was performed for all participants.

### Outcome measures

#### Primary outcome

PFM recruitment and progress from 24–30 to 36–38 weeks of gestation analyzed by the normalized root mean square (RMS) during rest-activity, fast and hold pelvic floor muscle contractions.

#### Secondary outcome

Clinical data related to parity, presence of previous diseases, age, gestational week, and glycemic levels of pregnant women receiving prenatal care in the public health care system were obtained from hospital records.

### Data analysis

**Fig 2:**
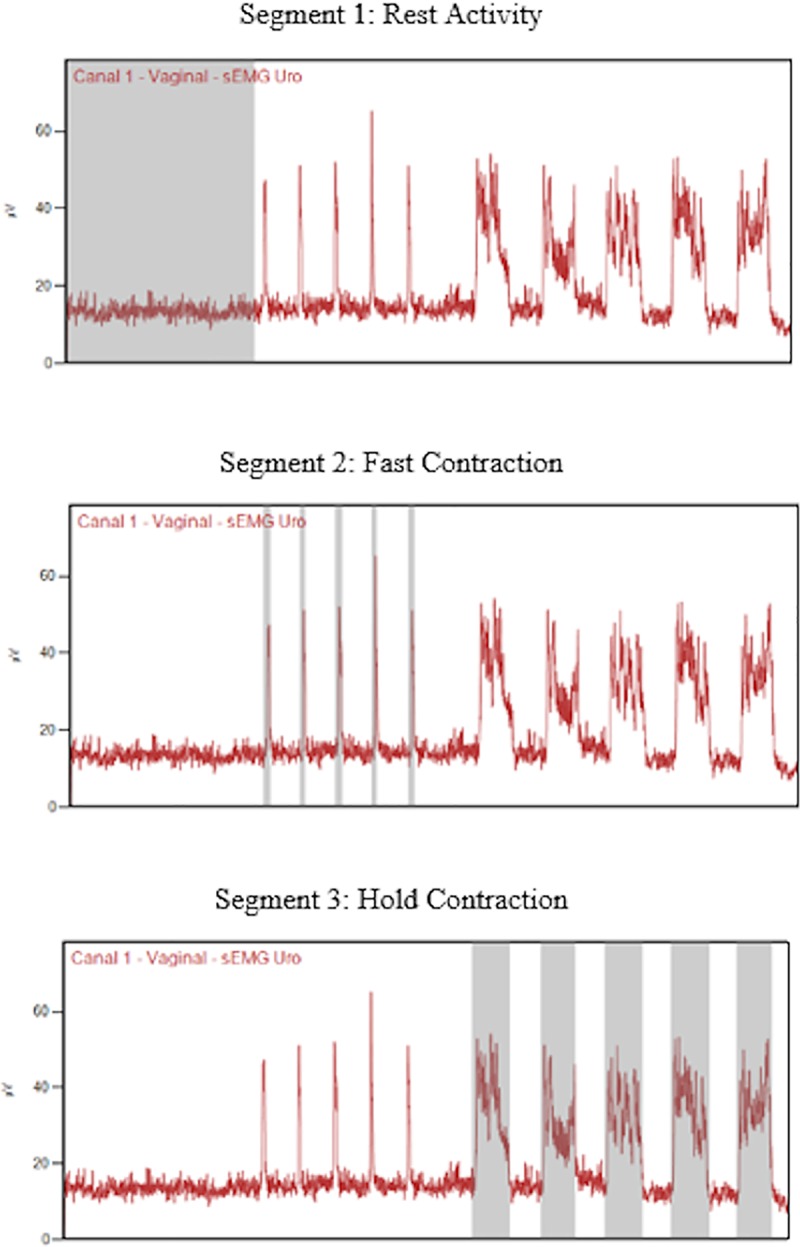
Hanning window procedure for rest activity and fast and hold contractions.

The raw signal of the EMG recording data was processed by using MiotecSuite software by an examiner blinded to the women’s clinical data. The electrical data of the recruitment root mean square (RMS) from the period of rest-activity was obtained by using Hanning window processing of the duration of the rest-activity period. The five fast and five hold contractions, separately, were performed by using Hanning window processing and selecting the most stable period, which was from the beginning of the contraction, identified visually as the point where the EMG activity clearly deviated from the baseline, and the end of the contraction, where the EMG activity returned to baseline. After this process, calculation of each RMS arithmetic mean of the fast and hold contractions was performed to determine a mean single value for each contraction type (Fig 2).[22] To normalize the EMG recruitment signal, we used the maximal fast contraction amplitude (RMS) chosen from among the 5 fast contraction values at 24–30 weeks of gestation because that was considered to be base data for analysis of changes in PFM activity (arithmetic mean of fast or hold contraction divided to maximal fast contraction). [22]

### Statistical methods

The Chi-square test or Fisher's exact test was applied for nominal data. Non-parametric tests were used; The Wilcoxon test was used to compare matched samples. The Mann–Whitney U test was applied to compare progress of PFM activity between groups. Delta calculation was performed between 24–30 weeks of gestation and 36–38 weeks of gestation (Δ = 36/38−24/30 weeks of pregnancy) to evaluate the changes this cohort two points and we called “progress” of PFM activity. The delta values of GDM and NG groups were compared. Quantile regression was used to examine the impact of GDM presence on progress of rest-activity, fast and hold contration from 28–30 to 36–38 weeks of gestation. *P* values < 0.05 were considered as indicating statistical significance.

## Results

### Flow of participants, therapists, centres through the study

**Fig 3:**
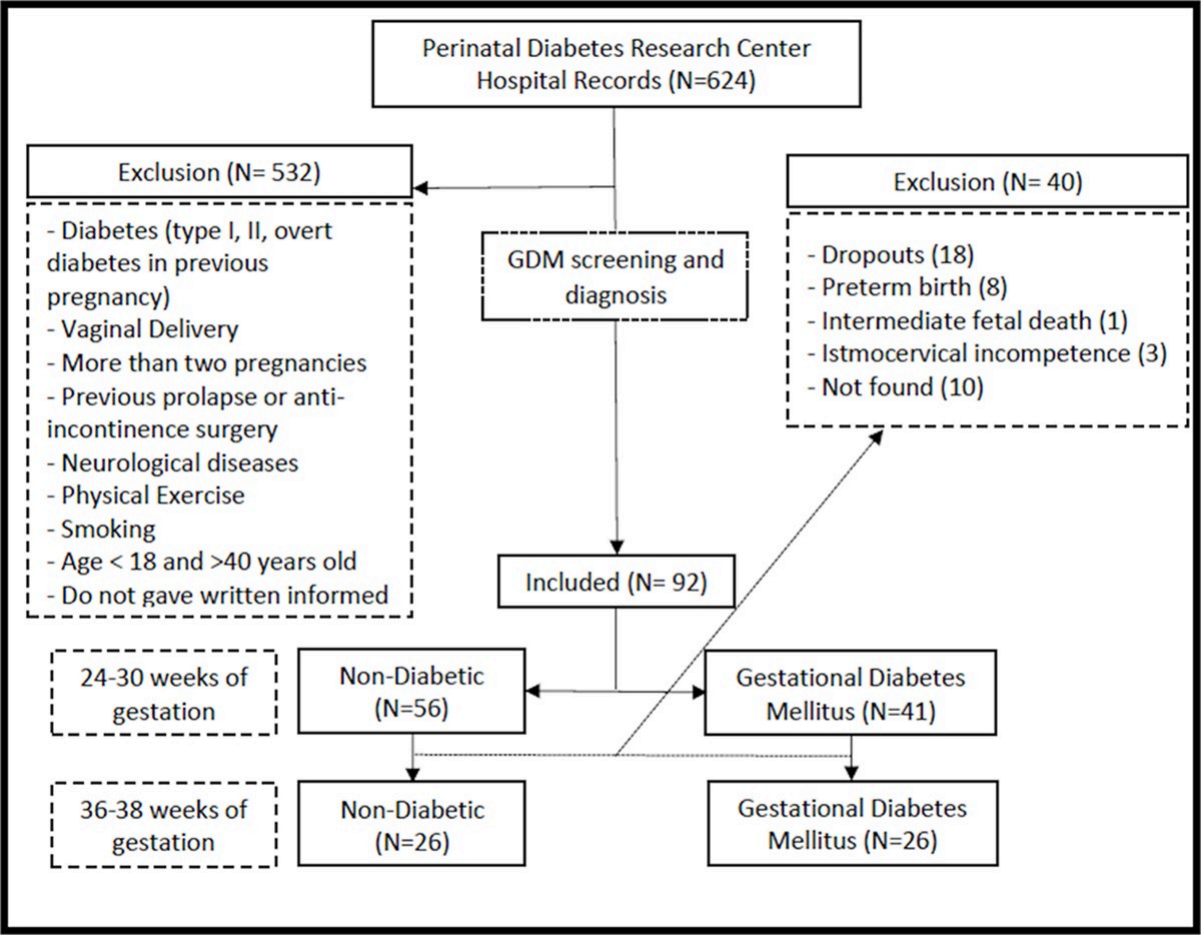
Flow diagram of GDM women’s screening, diagnosis, enrollment and follow-up analysis.

The flow chart in Fig 3 illustrates the number of women examined at each time point and the reasons for dropout. Amongst all included participants (n = 92) initially allocated, 56 women were in normoglycemic group and 41 in GDM group. Of the 56 normoglycemic women evaluated at 24–30 weeks of gestation, 26 were evaluated at 36–38 weeks of gestation. From 41 GDM women evaluated at 24–30 weeks of gestation, 26 were assessed at 36–38 weeks of gestation.

Table 1 shows that maternal age was paired between groups. Concerning that the window to PFM assessment was anytime between 24 to 30 weeks of gestation and to reassessment was 36 to 38 weeks of gestation, both groups showed similar gestational ages in two moments of the study. With respect to BMI, both groups showed similar characteristics during cohort study. The parity and modality of previous delivery were collected and similar percentages of C-section delivery was similar in both groups. Concerning the glucose tolerance test values, the main changes occur at fasting and 1 and 2 hours after OGTT, as expected. These similarities guarantee sample homogeneity. Table 2 shows a transversal analysis between groups at 24–30 and 36–38 weeks of gestation, and demonstrates no differences between groups.

**Table 1:** Baseline characteristics of ND and DMG group.

Variables	ND (n = 26)	GDM (n = 26)	P*
	Median (Min,Max)	Median (Min,Max)	
Age (years)^1^	29 (19,39)	29 (18,40)	.*826*
Gestational age median at 24–30^1^	27 (24,29)	27 (24,30)	.*477*
Gestational Age median at 36–38^2^	37 (36,38)	36 (36,38)	.*170*
BMI^3^ (kg/m2) at 24–30^1^	27.1 (21.2,32.9)	27.9 (20.4,38)	.*297*
BMI^3^ (kg/m2) at 36–38^2^	28.6 (22.4,34.1)	29.1 (22.5,39.4)	.*510*
Prior cesarean delivery^1^	7 (27%)	10 (23%)	.*749*
Fasting OGTT—(mg/dL)	75.5 (64,86)	86.5 (69,124)	*<* .*001*
1 hour OGTT	115 (72,149)	137 (82,211)	.*002*
2 hour OGTT	103.5 (69,143)	144 (72,182)	*<* .*001*

Data are the median (minimum, maximum) range or n (%)

^1^ evaluation at 24–30 weeks of gestation

^2^ evaluation at 36–38 weeks of gestation

^3^ BMI, body mass index.

^4^ OGTT, 75 g Oral Glucose Tolerance Test.

**Table 2:** Comparison between normalized root mean square (RMS) values from electromyography activity of pelvic floor muscles (PFM) in rest, fast contraction, and hold contraction from non-diabetic (ND) and gestational diabetes mellitus (GDM) groups at 24–30 and 36–38 weeks of gestation.

Variables	ND (n = 26)	GDM (n = 26)	P*
	Median (Min,Max)	Median (Min,Max)	
**24–30 weeks of gestation**			
Rest-activity^1^	0.23 (0.04,0.89)	0.24 (0.10,–0.84)	.*784*
Fast contraction^1^	0.66 (0.08,−1.89)	0.60 (0.21,0.99)	.*464*
Hold contraction^1^	0.70 (0.07,2.16)	0.57 (0.14,5.85)	.*884*
**36–38 weeks of gestation**			
Rest-activity^2^	0.29 (0.05,1.66)	0.19 (0.02,0.93)	.*092*
Fast contraction^2^	0.64 (0.10,2.05)	0.44 (0.12,2.52)	.*305*
Hold contraction^2^	0.70 (0.1,3.10)	0.41 (0.12,5.42)	.*213*

Data are the median (minimum, maximum) range.

^1^ evaluation at 24–30 weeks of gestation

^2^ evaluation at 36–38 weeks of gestation.

The normalized RMS values of PFM activity are shown in Table 3 demonstrates the delta changes from 24–30 to 36–38 weeks of gestation between groups called progress. Intragroup differences are present only in GDM group. GDM group decreases rest-activity from 0,24 at 24–30 weeks of gestation to 0,19 at 36–38 weeks of gestation (*P* = 0.041); the same occurs with the PFM recruitment in hold contraction 0,57 at 24–30 weeks of gestation versus 0,41 at 36–38 weeks of gestation (*P* = 0.049), although no differences during fast contractions were detected in GDM. Related to the progress of PFM activity between groups from 24–30 to 36–38 weeks of gestation, the results showed that GDM decreases PFM activity at rest and hold contractions instead of NG group maintaining the PFM activity. There was decrease in rest activity in GDM group around -6% from 24–30 to 36–38 weeks of gestation, while in NG group there was an increase of 1% between gestational ages (*P* = 0,004). The outcome measures were PFM recruitment during 24–30 and 36–38 weeks of gestation analyzed by the normalized root mean square (RMS) during rest-activity, fast and hold pelvic floor muscle contraction. Concerning fast contractions, no significant difference was detect, nevertheless the data behavior follows the tendency to decrease PFM activity in DMG and increase in NG (*P* = 0.194). In hold contractions, GDM group decreases PFM activity -6% whereas NG group maintain and even increase 4% PFM activity between two points.

**Table 3:** Progress and analysis intragroup of normalized Root Mean Square (RMS) Values From Electromyography Activity of Pelvic Floor Muscles (PFM) in Rest, Fast Contraction and Hold Contraction of the non-diabetic (ND) and Gestational Diabetes Mellitus (GDM) at 24–30 and 36–38 weeks of gestation.

		24–30 WG	36–38 WG	P*	Progress***	P**
Rest Activity	GDM (26)	0.24 (0.10,–0.84)	0.19 (0.02,0.93)	.041	−0.06 (−0.45,0.12)	.*042*
	ND (26)	0.23 (0.04,0.89)	0.29 (0.05,1.66)	.104	0.01 (−0.13,0.78)	
Fast Contraction	GDM (26)	0.60 (0.21,0.99)	0.44 (0.12,2.52)	.304	−0.06 (−0.43,1.52)	.*534*
	ND (26)	0.66 (0.08,−1.89)	0.64 (0.10,2.05)	.751	0.02 (−0.33,1.06)	
Hold Contraction	GDM (26)	0.57 (0.14,5.85)	0.41 (0.12,5.42)	.049	−0.06 (−3.47,0.53)	.*044*
	ND (26)	0.70 (0.07,2.16)	0.70 (0.1,3.10)	.571	0.04 (−0.43,1.46)	

WG = weeks of gestation; Data are the median (minimum, maximum) range.

* Analyses intragroup from 24–30 to 36–38 weeks of gestation

** Progress from 24–30 to 36–38 weeks of gestation

***Progress = (36/38–24/30 weeks of pregnancy).

According to regression analyses presented in Table 4, GDM group when compared to NG group during rest-activity progress presented a β = -0.074 (IC 95%: -0.115; -0.033) and GDM presence explain 6% of the model (r^2^ = 0.069). The same characteristics occurred in hold contraction progress the GMD group showed a β = -0.386 (IC 95%: -0.726; -0.046) when compared to NG group and the proposed model was able to explain 7% of the event. These data help us to identify GDM association with the decrement of rest activity and hold contraction from to 24–30 to 36–38 weeks of gestation.

**Table 4:** Quantile regression of PFM recruitment progress from 24–30 to 36–38 weeks gestation during rest-activity, fast and hold contractions.

Variables	β	95% C.I.	P	r^2^
Rest Activity Progress*	-0.074	-0.115; -0.033	0.001	0.069
Fast Contraction Progress*	-0.023	-0.223; 0.176	0.815	0.004
Hold Contraction Progress*	-0.386	-0.726; -0.046	0.027	0.076

*Progress = (36/38–24/30 weeks of pregnancy). CI = confidence interval.

**Fig 4:**
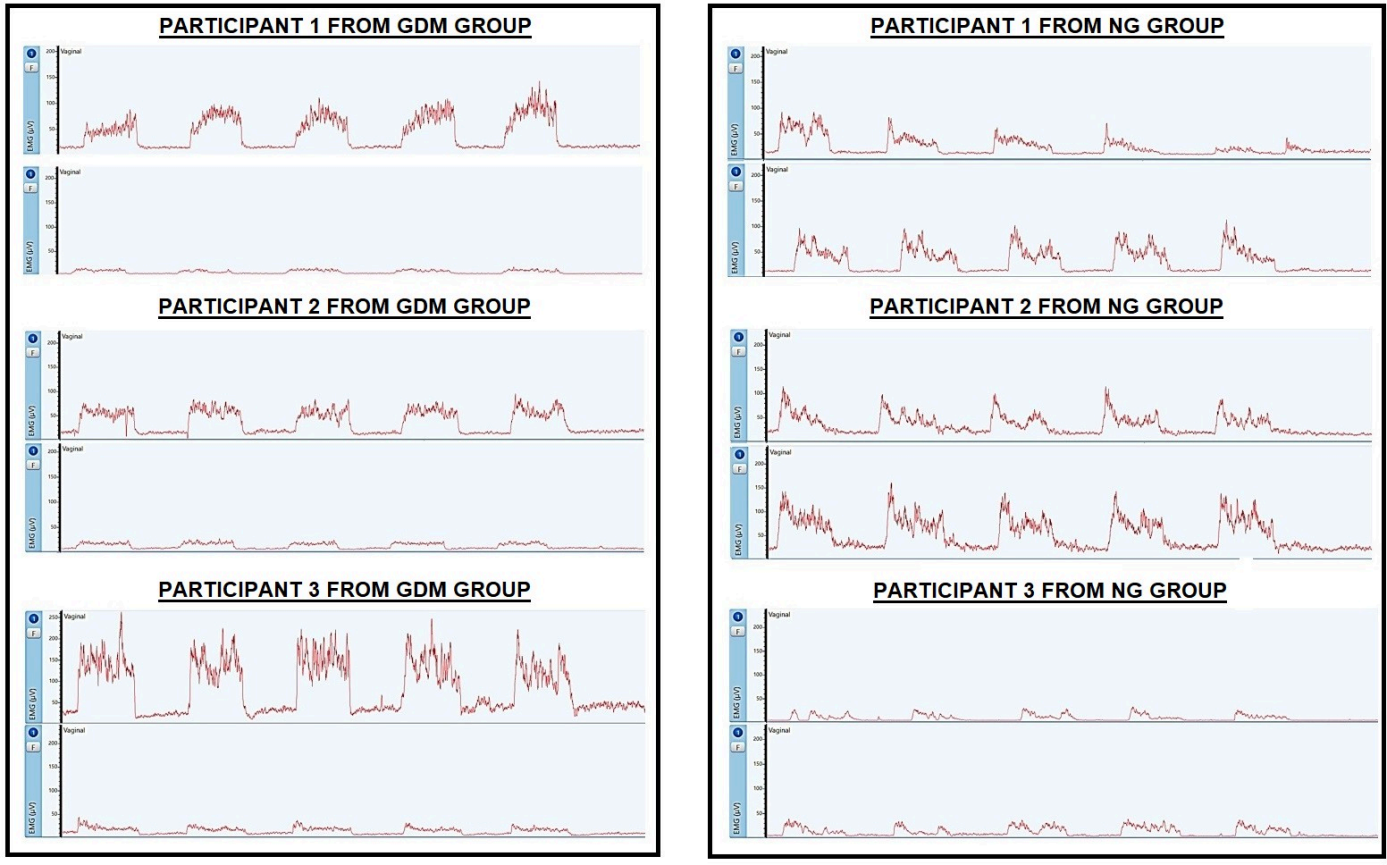
EMG RMS characteristics from 24–28 to 36–38 weeks of gestation of different 3 participants from DMG and NG group.

The EMG results presented before were performed by normalization process due to it is a gold standard to compare individuals. Although, Fig 4 illustrate the EMG RMS characteristics from 24–28 to 36–38 weeks of gestation of 3 different participants from each group to offer an overview of no normalized data in a qualitative analyses.

## Discussion

We set out to investigate and compare PFM activity in GDM women at two different gestational ages and thereby clarify the progression from 24–30 to 36–40 weeks of gestation. We found significant changes in PFM recruitment during rest-activity and hold contraction in GDM group who decreased function from 24–30 to 36–38 weeks of. This recruitment behavior and the homogeneity of baseline characteristics (Table 1) may suggest that GDM contributes for changes on PFM activity during rest and hold contraction from second to third trimester.

PFM rest-activity and hold contractions are important as these muscles are involved in postural stability, in maintenance of intra-abdominal pressure, and for mechanical support of the pelvic organ.[23] Pregnancy is associated with a progressive rise in intra-abdominal pressure and GDM pregnancies complicated by fetal macrosomia contribute to intense increase in maternal intra- abdominal pressure.[24]

A previous study showed that PFM rest-activity in non-pregnant insulin-resistant participants decreased compared to control group, which is consistent with our results.[14] There is a need to maintain lower basal activity even at rest because PFM is responsible for maintaining resting maximal urethral closure pressure, and when the ability to contract PFM is impaired, the maximal urethral closure pressure decreases by 70%–80%, which can lead to PFMD.[25,26] Decreased PFM activity in GDM could predispose pregnant women to develop PFMD, which is consistent with our clinical study that showing that even after 2 years postpartum women diagnosed with GDM presented higher urinary incontinence rates and PFMD.[27]

In addition, rest basal activity is a state preceding functional activation of PFM. In GDM, the decrement of PFM activity can be difficult to adjust recruitment for challenging functions because women with symptoms of PFMD show a delay in PFM contractions, in response to an increase in intra-abdominal pressure, which suggests that this delay is possibly influenced by insufficient preparatory recruitment transitioning from rest-activity to functional PFM activity.[26]

A previous study in non-pregnant insulin-resistant participants showed less recruitment of PFM during maximal voluntary contraction compared to control groups, although all procedures and the duration of contraction were not mentioned making comparison with our results difficult.[14]

Metabolic, neural, and muscular systems could be involved in lower rest-activity and hold contractions, and EMG has indicated that these systems are disturbed. Morphological changes in rat urethral muscles with mild diabetes can explain lower PFM activity by the lower fiber diameters than those in the NG group, colocalization of fast and slow fibers, and a decrease in the ratio of fast to slow fibers, which are characteristic of muscles with decreased capacity to originate normal electrical signals.[6]

In addition to myopatic disorders in GDM, there are further changes in the extracellular matrix that involve the presence of interfibrillar and intermyofibrillar collagen found in diabetic pregnant rats.[5] The connective tissue has an important role in muscle structure and function because it involves muscle fibers to guarantee tension, providing good performance. The fibrosis process could restrict the slippage of muscle fibers.[28] Similarly, lipids and granules of intramuscular mitochondria misalign myofibrils and make sliding of fibers difficult.[5,7]

Regarding substrates of skeletal muscle, metabolism studies have shown a decrease in the oxygen supply to muscles in GDM. These characteristics interfere with the ability to maintain contraction for a prolonged period by limiting the ability of the main substrate to hold the contraction.[29,30]

The changes were predominant in tasks involving slow fiber activation to fast fibers. We hypothesized the tendency of GDM to affect major slow fiber types and led us to suspect that atrophy of urethral striated muscle is probably involved in neural changes. Myelin abnormalities are associated with diabetes, so we suggest that this characteristic is caused by damage in smaller motor units that present thicker myelin sheath than those of fast fibers, therefore becoming the first to be affected.[31] Neuromuscular transmission failure and possibly pathological alterations in muscle metabolism have been attributed to decreases in muscle activity.[32]

One of the strengths of this study was the analysis of progress between pregnancy stages. This prospective cohort study has a translational source and confirms our previous experimental models with clinical data.[5,6] Fig 4 illustrated a no-normalized data to provide an overview of EMG RMS signal picture to help clinical professional to have a practical view of EMG from 24–28 to 36–38 weeks of gestation between groups, it is evident the difference in an qualitative analyses. However, technically is important the normalization procedure adopted to minimizes external artefacts and contributes reliable EMG data. Our data showed differences even after normalization procedure a fact that support our data differences.

A possible limitation of our study is the absence of pre-pregnancy clinical data. In our study, each women was their own control, but the baseline was just before the GDM diagnoses so we were only able to look at associations between GDM and PFM EMG at different time points during pregnancy. Another limitation is the difficulty to include women at the beginning of pregnancy in the first trimester, which could have limited our ability to obtain more significant results in the DMG and NG groups. [33]

Our higher dropouts rates was other negative limitation, we faced difficulty to maintain pregnant during the 2 study points, we believe that even though EMG exam is a safe and comfortable procedure the fact of pregnant need to spend around 40 minutes additionally of the prenatal care and the invasive procedures could contribute to this higher rate. Another study with pelvic exams reported the same difficult to maintain participant during cohort and corroborate our study. [34]

Although glycemic control was made clinically, another limitation of study is no availability of the blood glucose concentrations following a diagnosis of GDM. [33] In the other hand, previous study showed that women with GDM diagnosis and treated with diet and insulin administration had significant negative effect on maternal and fetal outcomes. [2]

This was the first study to demonstrated directly changes in PFM during pregnancy complicated by GDM, knowledge of the neuromotor behavior of PFM is of paramount importance for the training and reorganization of motor planning in pregnancy.[35,36] This investigation contributes to the understanding of PFM recruitment in GDM women at two time points of gestation. There was same methodological limitation that limited our conclusions, so it is important that the next studies provide more information about data that was presented as limitation in this present study.

In conclusion, the results of this study demonstrate that GDM group present a progressive decrease in EMG-PFM activity in rest-activity and hold contractions from 24–30 to 36–38 weeks of gestation.

## Supporting information

S1 Fig Database. (PDF)
