# Supplementary material for: Negative impact of gestational diabetes mellitus on progress of pelvic floor muscle electromyography activity: Cohort study
Source: PLoS One. 2019 Nov 7;14(11):e0223261. doi: 10.1371/journal.pone.0223261 (PMC6857854; doi:10.1371/journal.pone.0223261)
Supplement: S1 Fig — (PDF) [file pone.0223261.s001.pdf]

CODN	GPO	ID	IND_CESPRE	GLICJEJ	TTGO0	TTGO60	TTGO120	IMC1
1	0	19	0	91	72	72	95	28,5
2	0	33	1	88	78	143	110	25,3
3	0	25	0	86	71	124	127	32,3
4	0	22	0	70	64	126	88	21,6
5	0	30	1	85	78	115	111	31,6
6	0	38	0	76	79	72	89	32,8
7	0	21	0	81	71	126	88	32,9
8	0	29	0	87	75	97	78	26,5
9	0	32	1	89	76	126	127	27,3
10	0	28	0	74	73	76	69	23,5
11	0	39	0	65	71	115	135	21,2
12	0	33	1	80	81	154	143	27,7
13	0	32	0	89	73	163	111	24,7
14	0	25	0	75	81	115	127	30,0
15	0	33	1	81	76	102	102	24,2
16	0	22	0	86	71	113	98	30,3
17	0	24	0	71	74	92	76	27,2
18	0	23	0	66	86	124	117	22,2
19	0	25	0	80	81	126	143	31,6
20	0	31	1	91	78	113	97	29,3
21	0	22	0	76	81	113	104	26,44
22	0	29	0	89	73	163	111	26,3
23	0	33	1	79	72	115	103	27,0
24	0	22	0	75	78	76	70	25,3
25	0	30	0	73	71	76	104	22
26	0	29	0	84	79	101	69	31,1
27	1	21	0	96	75	137	159	27,2
28	1	27	0	85	124	197	133	25,0
29	1	25	0	92,5	82	179	156	38,0
30	1	27	0	95,7	92	82	103	25,1
31	1	19	0	91	78	177	153	30,1
32	1	32	0	94	94	106	116	37,4
33	1	20	0	76	92	211	147	20,4
34	1	29	1	80	78	167	182	32,7
35	1	30	0	98	88	211	147	21,7
36	1	18	0	77	81	113	162	21,6
37	1	23	0	85	100	133	157	32,4
38	1	29	0	79	103	136	144	27,8
39	1	16	0	75	92	88	78	25,3
40	1	22	0	92	84	180	144	27,0
41	1	36	0	76	83	183	131	22,2
42	1	30	1	100	100	136	172	35,8
43	1	28	0	85	69	150	162	23,6
44	1	41	1	81	119	133	72	26,6
45	1	33	1	76	76	191	179	22,0
46	1	36	1	83	98	186	72	36,6
47	1	29	0	94	91	124	117	34,4
48	1	30	0	85	75	137	159	28,8
49	1	27	0	87	96	108	103	28,1

50	1	29	0	93	78	89	123	34,2
51	1	35	0	99	85	174	124	30,8
52	1	31	1	64	83	102	96	37,6

SEM1	IMC2	SEM2	Rep1_N3	Rep2_N3	V_Rep_N3	R1_N3	R2_N3	V_R_N3
26	30,5	36	0,08	0,11	0,02	0,58	0,25	-0,33
28	27,9	36	0,11	0,10	-0,01	0,58	0,62	0,04
25	33	37	0,39	0,44	0,05	0,55	0,57	0,02
26	24,5	38	0,40	0,30	-0,10	1,35	1,44	0,09
26	33,9	37	0,83	0,73	-0,10	0,30	0,31	0,02
28	33,0	37	0,51	0,55	0,04	1,08	0,80	-0,28
28	34,0	36	0,04	0,05	0,00	0,17	0,18	0,02
27	28,0	36	0,20	0,18	-0,02	0,93	1,99	1,06
28	30,9	36	0,67	0,55	-0,13	1,64	1,98	0,34
29	24,8	37	0,16	0,16	0,00	0,08	0,10	0,02
28	22,4	37	0,04	0,05	0,01	1,29	1,17	-0,12
24	29,8	36	0,89	1,66	0,78	0,58	0,62	0,04
24	27,6	38	0,07	0,19	0,12	0,12	0,15	0,03
29	32,7	36	0,51	0,53	0,02	0,95	0,71	-0,23
26	25,6	36	0,04	0,05	0,01	0,94	0,90	-0,04
26	31,6	37	0,87	0,88	0,01	1,35	1,17	-0,18
27	28,7	37	0,16	0,21	0,05	0,73	0,57	-0,16
28	23,7	37	0,04	0,07	0,03	0,23	0,34	0,11
26	34,1	37	0,36	0,59	0,23	0,18	0,34	0,16
29	30,2	37	0,18	0,36	0,18	0,36	0,16	-0,20
27	28,3	37	0,20	0,22	0,02	0,08	0,12	0,04
26	27,5	36	0,33	0,39	0,06	1,45	1,33	-0,12
28	28,6	35	0,11	0,38	0,27	1,89	2,05	0,16
26	27,1	37	0,39	0,28	-0,11	1,17	1,07	-0,10
24	25,07	37	0,41	0,42	0,01	0,44	0,66	0,23
24	32,4	36	0,25	0,17	-0,08	1,79	1,57	-0,22
28	28,5	36	0,39	0,13	-0,27	0,78	0,80	0,03
24	26,0	37	0,23	0,17	-0,06	0,21	0,35	0,14
24	39,1	36	0,31	0,19	-0,12	0,53	0,15	-0,37
28	25,0	36	0,14	0,18	0,04	0,58	0,85	0,28
25	34,7	37	0,17	0,04	-0,14	0,66	0,44	-0,21
28	38,7	36	0,73	0,28	-0,45	0,48	0,71	0,23
28	22,5	38	0,21	0,33	0,12	0,58	0,67	0,09
28	35,9	36	0,24	0,30	0,06	0,49	0,35	-0,14
27	23,0	38	0,25	0,06	-0,19	0,64	0,82	0,18
26	22,8	36	0,14	0,21	0,07	0,61	0,18	-0,43
26	33,6	36	0,11	0,14	0,04	0,49	0,53	0,04
28	28,0	36	0,38	0,49	0,11	0,62	0,66	0,03
29	26,4	36	0,10	0,02	-0,08	0,56	0,65	0,09
28	30,4	36	0,14	0,13	-0,01	0,60	0,59	-0,01
27	23,6	36	0,84	0,93	0,09	0,51	0,40	-0,11
26	39,4	37	0,18	0,08	-0,10	0,62	0,31	-0,31
29	25,3	36	0,16	0,11	-0,05	0,99	2,52	1,52
26	27,7	36	0,22	0,18	-0,04	0,66	0,33	-0,33
26	23,7	36	0,13	0,05	-0,07	0,51	0,36	-0,15
25	38,2	36	0,48	0,38	-0,10	0,66	0,76	0,10
27	35,8	37	0,29	0,36	0,07	0,53	0,12	-0,41
27	29,7	36	0,72	0,28	-0,44	0,62	0,42	-0,19
28	28,2	37	0,59	0,20	-0,40	0,62	1,58	0,96

30	38,3	36	0,22	0,16	-0,06	0,61	0,43	-0,18
25	32,1	36	0,27	0,38	0,11	0,64	0,27	-0,37
29	39,0	36	0,32	0,19	-0,13	0,48	0,30	-0,18

L1_N3	L2_N3	V_L_N3
0,48	0,14	-0,35
0,74	0,69	-0,05
0,59	0,63	0,03
1,09	1,46	0,38
0,37	0,29	-0,08
0,68	0,60	-0,09
0,12	0,17	0,06
0,79	2,24	1,46
2,00	1,84	-0,16
0,07	0,11	0,04
1,16	1,23	0,08
0,71	0,50	-0,20
0,11	0,12	0,01
1,19	0,76	-0,43
0,89	1,01	0,12
1,59	1,72	0,13
0,31	0,96	0,65
0,15	0,43	0,28
0,17	0,21	0,05
0,34	0,18	-0,16
0,12	0,11	-0,01
1,83	1,42	-0,40
2,16	3,10	0,94
1,59	1,18	-0,41
0,39	0,70	0,31
1,46	2,32	0,86
0,15	0,19	0,04
0,19	0,30	0,11
0,34	0,16	-0,18
0,51	0,57	0,06
0,66	0,38	-0,29
0,84	0,12	-0,73
0,56	0,56	-0,01
0,77	0,74	-0,03
0,32	0,60	0,28
0,69	0,20	-0,49
0,27	0,23	-0,03
1,21	1,74	0,53
0,43	0,24	-0,18
1,07	1,06	-0,01
5,85	5,42	-0,43
0,78	0,44	-0,34
0,16	0,24	0,08
0,45	0,24	-0,20
0,30	0,22	-0,08
1,55	1,55	0,00
3,99	0,52	-3,47
0,14	0,18	0,04
0,26	0,60	0,34

1,09	0,53	-0,56
2,26	1,04	-1,22
0,57	0,32	-0,25
